# Nuclear Localization of HBD-1 in Human Keratinocytes

**Published:** 2007-08-24

**Authors:** Roger J. Bick, Brian J. Poindexter, L. Maximilian Buja, Carl H. Lawyer, Stephen M. Milner, Satyanarayan Bhat

**Affiliations:** Department of Pathology and Laboratory Medicine, University of Texas Medical School at Houston, Houston; The Institute for Plastic and Reconstructive Surgery, Southern Illinois University School of Medicine, Springfield; Johns Hopkins Burn Center, Johns Hopkins University, Baltimore, MD

## Abstract

**Objective:** Human defensins and cathelicidins are a family of cationic antimicrobial peptides (AMPs), which play multiple roles in both innate and adaptive immune systems. They have direct antimicrobial activity against several microorganisms including burn pathogens. The majority of components of innate and adaptive immunity either express naturally occurring defensins or are otherwise chemoattracted or functionally affected by them. They also enhance adaptive immunity and wound healing and alter antibody production. All mechanisms to explain multiple functions of AMPs are not clearly understood. Prior studies to localize defensins in normal and burned skin using deconvolution fluorescence scanning microscopy indicate localization of defensins in the nucleus, perinuclear regions, and cytoplasm. The objective of this study is to further confirm the identification of HBD-1 in the nucleus by deconvolution microscopic studies involving image reconstruction and wire frame modeling. **Results:** Our study demonstrated the presence of intranuclear HBD-1 in keratinocytes throughout the stratum spinosum by costaining with the nuclear probe DAPI. In addition, HBD-1 sequence does show some homology with known cationic nuclear localization signal sequences. **Conclusion:** To our knowledge, this is the first report to localize HBD-1 in the nuclear region, suggesting a role for this peptide in gene expression and providing new data that may help determine mechanisms of defensin functions.

Antimicrobial peptides (AMPs), including both defensins and cathelicidins, are part of the innate immune system, which provides the first line of defense against a wide spectrum of common burn and wound pathogens.^1^–^5^ In the past few years, multiple functions of AMPs have been identified.^6^–^9^ They are chemotactic for neutrophils, monocytes, immature dendritic cells, memory T cells, and mast cells, and play a role in recruiting them. They also alter transcriptional responses in macrophages, induce degranulation of mast cells, and stimulate wound vascularization and re-epithelialization in wound healing, as well as altering antibody production, thus linking innate and adaptive immunity. In addition, LL-37, a human cathelicidin, possesses the ability to bind and neutralize gram-negative lipopolysaccharide (LPS), decrease nitric oxide/inducible nitric oxide synthase (NO/iNOS) production, and reduce septic shock.^10^ The mechanisms by which AMPs are able to exhibit such multiple functions are not fully understood.

Human defensins are cationic antimicrobial peptides with molecular weights of 4 to 5 kDa, containing a conserved motif of 6 cysteines linked by 3 disulfide bonds. On the basis of their size and pattern of disulfide bonding, human defensins are classified into α and β categories, and are expressed in various cell types.^11^–^20^ Out of 6 α-defensins, human neutrophils express 4 of them, viz, human neutrophil peptides (HNP1-4), whereas the other 2 α-defensins (HD-5 and HD-6) are abundantly expressed in Paneth cells of the small intestine and epithelial cells of the female urogenital tract. The human β-defensins (HBDs; HBD-1-4) are widely expressed in epithelial cells of multiple organs including skin. The β-defensins are less abundant, and their production by epithelial cells is subject to regulation by a variety of inflammatory and microbial stimuli.^11^–^20^

In our previous work, we have localized AMPs in burned and normal skin to specific cell types and areas of the epidermis and dermis, using deconvolution fluorescence microscopy.^21^, ^22^ During these current studies, we observed evidence of an intranuclear localization of HBD-1. A recent study conducted by Bandholtz et al^23^ demonstrated uptake of LL-37 by monocyte-derived dendritic cells and subsequent localization to both cytoplasmic and nuclear elements. Also, LL-37 was shown to mediate uptake of DNA plasmids into cytoplasmic and nuclear location of mammalian cells.^24^ Since LL-37 and HBD-1 share some similar properties, we have extended our work in the current study to focus on nuclear localizations of HBD-1 in normal skin. This new finding may have implications in gene expression and shed light on the multiple roles of defensins.

## MATERIALS AND METHODS

Patients admitted to the Regional Burn Center at Memorial Medical Center, Springfield, IL, or the Johns Hopkins Burn Center with 10% to 40% total body surface area burns were included in the study. Normal skin samples were obtained from remnants of split thickness skin autografts harvested during skin grafting procedures and were frozen in sucrose-based embedding media (O.C.T. Compound Tissue-Tek, Torrance, Calif). Ten-micrometer sections were cut in a microtome, floated onto 18-mm glass cover slips that had been coated with poly-l-lysine, and fixed with 3.7% paraformaldehyde. This was followed by 60-min incubation in goat serum and staining with appropriate probes and antibodies as previously described.^22^

### Deconvolution Microscopy

The stained sections were placed under Elvanol (an antifade, DuPont), and covered with a cover slip. The samples were scanned with an Applied Precision Delta Vision Scanning Microscope (Issaquah, Wash), fitted with an Olympus 1 × 70 microscope (Olympus America, Melville, NY). Image scans were acquired in a series at a step-size thickness of 0.2 μ m by a Photometries (Tucson, Ariz) PXL CCD camera. Image analysis was performed by transferring each data set to a Silicon Graphics workstation using SoftWoRxTM software (Applied Precision, Issaquah, Wash). All data sets were subjected to 5 deconvolution iterations and subsequently used for image reconstructions and modeling. Stacking each of the individual sections produces a 3-dimensional (3D) image on a 2-dimensional background, resulting in an image projection.^22^ Wire framing of the images is carried out as described previously to identify distinct colors of defined wavelength patterns for HBD-1 and DAPI.^25^

## RESULTS

**Figure 1:**
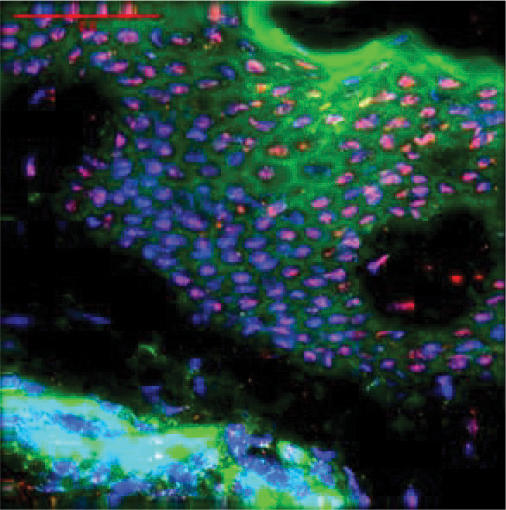
Localization of HBD-1 in normal skin by deconvolution fluorescence microscopy. HBD-1 was found homogeneous throughout the epidermis, in the nuclei of keratinocytes. (magnification bar = 50 μ m; blue = DAPI (nuclei), green = actin, and red = texas red tagged peptide antibody.)

**Figure 2:**
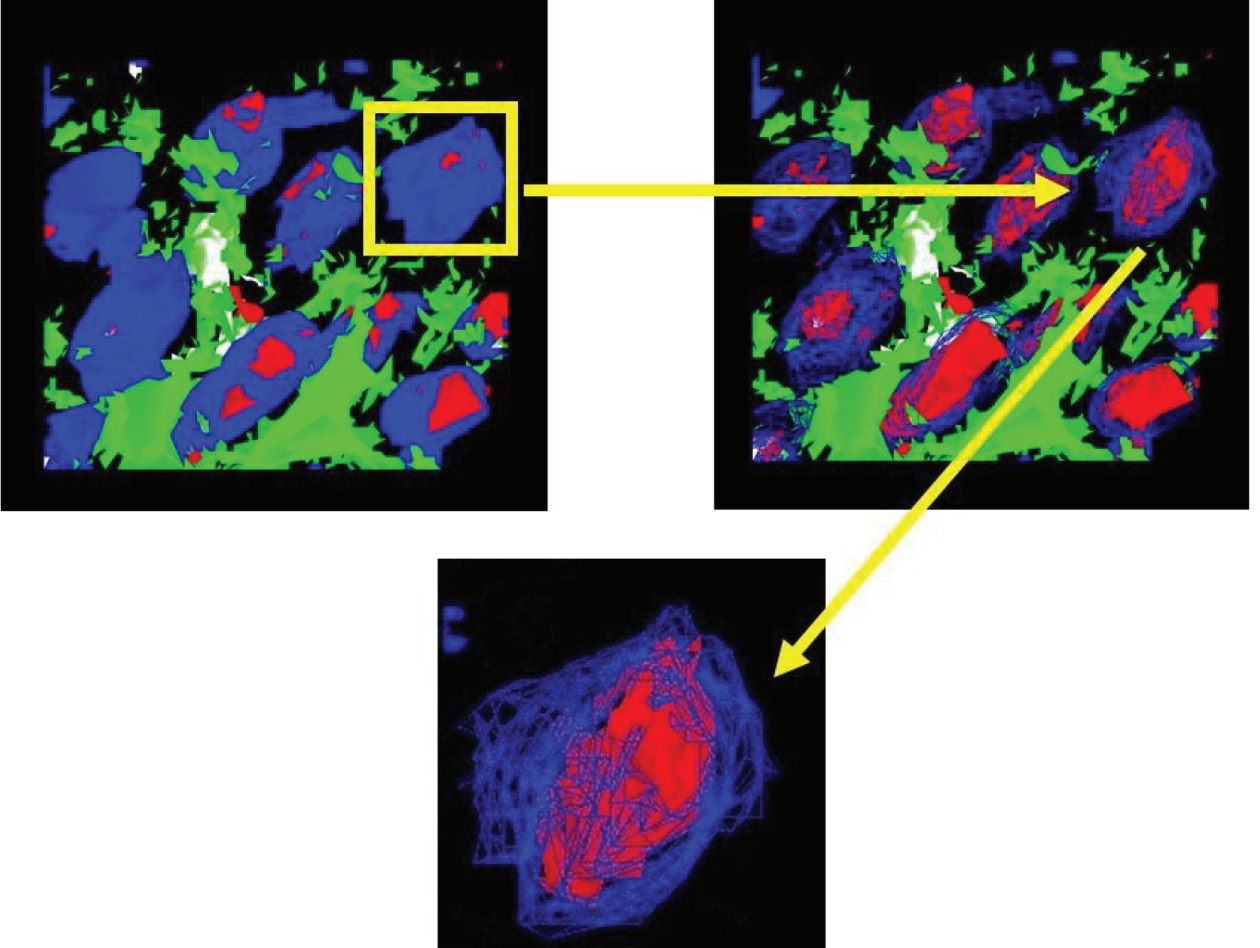
Deconvoluted, 3-dimensional rendition of multiple-stacked acquisitions of HBD-1 in the epidermis. A nucleus from the volume rendered image is then extracted and wire framed, allowing visualization of the HBD-1 within the nucleus, shown at a high magnification in the final panel (magnification × 4000).

The Figure 1 illustrates localization of HBD-1 in normal skin in which its presence was found homogeneously throughout the epidermis, contained in keratinocytes. By using the fluorescent nuclear probe DAPI, we noticed that HBD-1 appeared to be colocalized with the nuclei so; to further characterize this apparent nuclear, we stacked multiple deconvoluted (5 iterations) to generate an image representing some thickness of skin, and then modeled these stacked renditions to produce a 3D model that permitted rotation (Fig 2).

**Figure 3:**
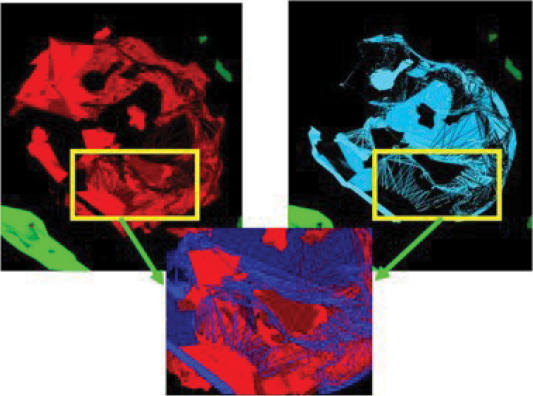
Three-dimensional volume rendition to demonstrate the 2-color channels (DAPI and Texas Red), and show intranuclear localization of HBD-1. The blue of the nucleus overlying the red of the antimicrobial peptide further confirms the intranuclear localization of HBD-1.

The extraction of a number of cells and nuclei from these 3D models and the application of fish-net mapping permitted us to visualize specific probes as distinct areas with no color-mixing (colocalization) and single color/channel imaging. It is apparent (Fig 2) that the red HBD-1 is within the nucleus, being encompassed by the blue of the DAPI. This finding is further confirmed in Figure 3, using a wire frame procedure, splitting the images into the 2-component probe channels, and applying color assignments.

**Figure 4:**
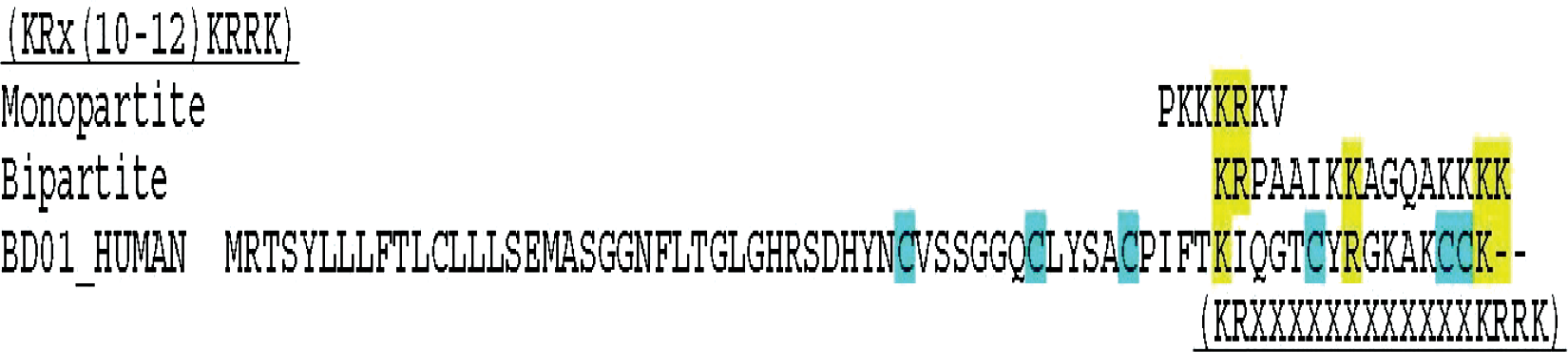
The sequences of HBD-1 is compared with nuclear targeting sequences of monopartite and bipartite proteins which is known to contain reported nuclear targeting sequence (KRx(10-12)KRRK). Yellow color indicates homology.

We attempted to detect nuclear targeting sequences in HBD-1. This was achieved using a database search engine (http://walnut.bioc.columbia.edu/srs71bin/cgi-bin/wgetz). HBD-1 show some homology with the nuclear targeting sequences (KRX(10-12)KRRK) of the known nuclear translocation proteins, monopartite and bipartite (Figure 4). This may indicate that defensins may be transported into the nucleus, possibly through the nuclear pore.

## DISCUSSION

This study demonstrates nuclear localization of HBD-1. Considering that both defensins and cathelicidins display multiple functions, we hope that current findings may help explain mechanisms for some of these functions. AMPs are able to differentially recognize and disrupt negatively charged bacterial cell membranes,^1^–^4^ while also being able to recruit many components of the innate and adaptive immune systems such as macrophages, neutrophils, antigen presenting dendritic cells, NK-cells, and T- and B-cells, by chemo attraction.^6^–^8^ It has been shown that the mechanisms of chemo-attraction and direct killing are independent of each other,^9^ the upregulation or release of AMPs by other cells at the site of injury helping recruit other innate immune components, while some components of both innate and adaptive immunity also produce defensins and cathelicidins. AMPs are implicated in wound healing, angiogenesis, gene expression, cell differentiation and migration, antigen presentation, and antibody production. AMPs affect intracellular signaling by diverse kinase pathways, alter chemokine and chemokine receptor gene expression, and also mediate and modulate cytokine release.^26^–^33^ Even though some of the mechanisms for these multiple functions have already been defined, we still need to know the other pathways involved in the actions of AMPs, and future studies may reveal these mechanisms, particularly as defensins have been shown to alter the expression of cytokines through an, as yet, undefined pathway.

Currently, there are 2 AMPs which have been localized to the nucleus, this study localizing HBD-1 in the nucleus of keratinocytes in skin, and the recent study by Bandholtz et al,^23^ localizing the LL-37 to the nuclei of monocyte-derived dendritic cells (MDDC). LL-37 also promotes nuclear transport of plasmid DNA in mammalian cells,^24^ and so taking these findings together, it is reasonable to hypothesize that AMPs enter the nucleus, possibly playing a role in the modulation of gene expression and/or protein synthesis.

The mechanism by which AMPs enter the nucleus has not been studied and is subject to speculation, although several possibilities exist. In eukaryotic cells, signal-mediated macromolecular transport between the nucleus and the cytoplasm is an integral part of many processes, such as gene expression, signal transduction, and cell-cycle progression. Even though molecules can enter the nucleus of actively dividing cells through nuclear membrane breaks during mitosis, the only portal of entry into the nucleus of nondividing cells is the nuclear pore complex (NPC). NPCs allow passive exchange of ions, small molecules, and small proteins (< 20 kDa), but restrict the passage of macromolecules to only those bearing appropriate nuclear localizing signal (NLS). Considering the small size of AMPs (4–5 kDa for defensins), the question arises as to whether NPCs do allow the passive transport of AMPs into the nucleus.

Another mechanism by which AMPs could enter the nucleus is based on AMP cationic sequence, a sequence that could constitute a nuclear localization and recognition signal and promote nuclear transportation. The directional transport of thousands of proteins and RNAs through the NPC in the course of normal cellular metabolism is mediated by a family of more than 20 proteins, the karyopherins.^34^–^37^ The import of proteins carrying a cationic NLS involves an adaptor protein karyopherin-α (Kapα), which in turn binds to karyopherin-β and interacts with the NPC for translocation into the nucleus. Karyopherin-β can also directly bind proteins for nuclear import. The sequence motifs for direct karyopherin-β translocation are not well defined, and are not necessarily cationic. HBD-1 shares some homology with known nuclear translocation signals of cationic monopartite and bipartite molecules. Kapα recognizes a variety of classical NLSs, such as the basic monopartite SV40 T antigen NLS (PKKKRKV), the more hydrophobic monopartite c-Myc NLS (PAAKRVKLD) and the bipartite nucleoplasmin NLS (VKRPAATKKAGQAKKKKLD).^38^ Although lacking a specific consensus sequence, the classical NLSs contain either 1 or 2 clusters of basic residues.

Polymers of basic amino acids [i.e poly (Lys)_16_ and poly (Arg)_16_] promote translocation of plasmid DNA into the nucleus. Many cationic peptides, other than those known to function as nuclear localizing motifs, appear to have a nuclear translocation ability regarding DNA plasmids. The ability of LL-37 to deliver plasmid DNA into mammalian cells has been demonstared.^24^ The physiologically relevant concentrations of LL-37, widely expressed in bone marrow and epithelial cells, protects plasmid DNA against serum nuclease degradation and efficiently targets DNA to the nuclear compartment of mammalian cells. Furthermore, mechanistic data indicate that LL-37-DNA complexes enter mammalian cells via endocytosis that involves noncaveolar lipid raft domains, as well as cell surface peptidoglycon.^24^ Currently, however, there have been no data demonstrating the ability of HBD-1 and other defensins to deliver plasmid DNAs.

One possible functional importance of nuclear localization of AMPs may be coupled to affecting gene expression.^26^ Even though there is no direct link that HBD-1 participates in gene expression, sufficient evidence exists to demonstrate that other AMPs, for example, HBD 2-4 and LL-37, alter gene expression. It has now been clearly demonstrated that AMPs alter antibody production in response to exposure to antigen.^6^–^8^ LL-37 triggers a transformation from monocytes to MDDC, which involves alteration in gene expression. It has also been reported that immature MDDCs (iMDDC) take up LL-37, which is predominantly localized not only to cytoplasmic locales but also to some extent to the nucleus. LL-37 also alters MDDC phenotype with increased expression of the antigen-presenting molecule HLA-DR and the costimulatory molecule CD86.^34^ MDDCs is known to express HBD-1. As it is known that AMPs acts as an adjuvant, it is quite reasonable to hypothesize that AMPs could influence gene expression responsible for overall alteration of antibody patterns. It is also interesting to note that B-cells are a site of defensin production and are chemo attracted by AMPs.^35^ Previous suggestions have been made that the action of AMP may be similar to that of lactoferrin, that has been shown to undergo translocation into lymphocyte nuclei, in order to bind DNA and activate transcription.^37^, ^41^ In addition, these AMPs could alter gene expression indirectly, without getting translocated to nucleus by other mechanisms involving cell surface receptor binding.

In summary, our observation, when taken together with other previously reported findings of the actions and mechanisms of AMPs, suggest possibilities for new roles for AMPs in the modulation of gene expression and/or protein synthesis. Further studies are required to elucidate these possibilities.
